# Supplementary material for: Klebsiella pneumoniae peptide hijacks a Streptococcus pneumoniae permease to subvert pneumococcal growth and colonization
Source: Commun Biol. 2024 Apr 8;7:425. doi: 10.1038/s42003-024-06113-9 (PMC11001997; doi:10.1038/s42003-024-06113-9)
Supplement: Supplementary file 2 — Supplementary Information [file 42003_2024_6113_MOESM2_ESM.pdf]

SUPPLEMENTARY INFORMATION

Klebsiella pneumoniae peptide hijacks a *Streptococcus pneumoniae* permease to subvert pneumococcal growth and colonization

Janine Lux, Hannah Portmann, Lucía Sánchez García, Maria Erhardt, Lalaina Holivololona, Laura Laloli, Manon F. Licheri, Clement Gallay, Robert Hoepner, Nicholas J. Croucher, Daniel Straume, Jan-Willem Veening, Ronald Dijkman, Manfred Heller, Denis Grandgirard, Stephen L. Leib, Lucy J. Hathaway

This file includes:

Supplementary Figures 1-8

Supplementary Tables 1-2

SUPPLEMENTARY FIGURES

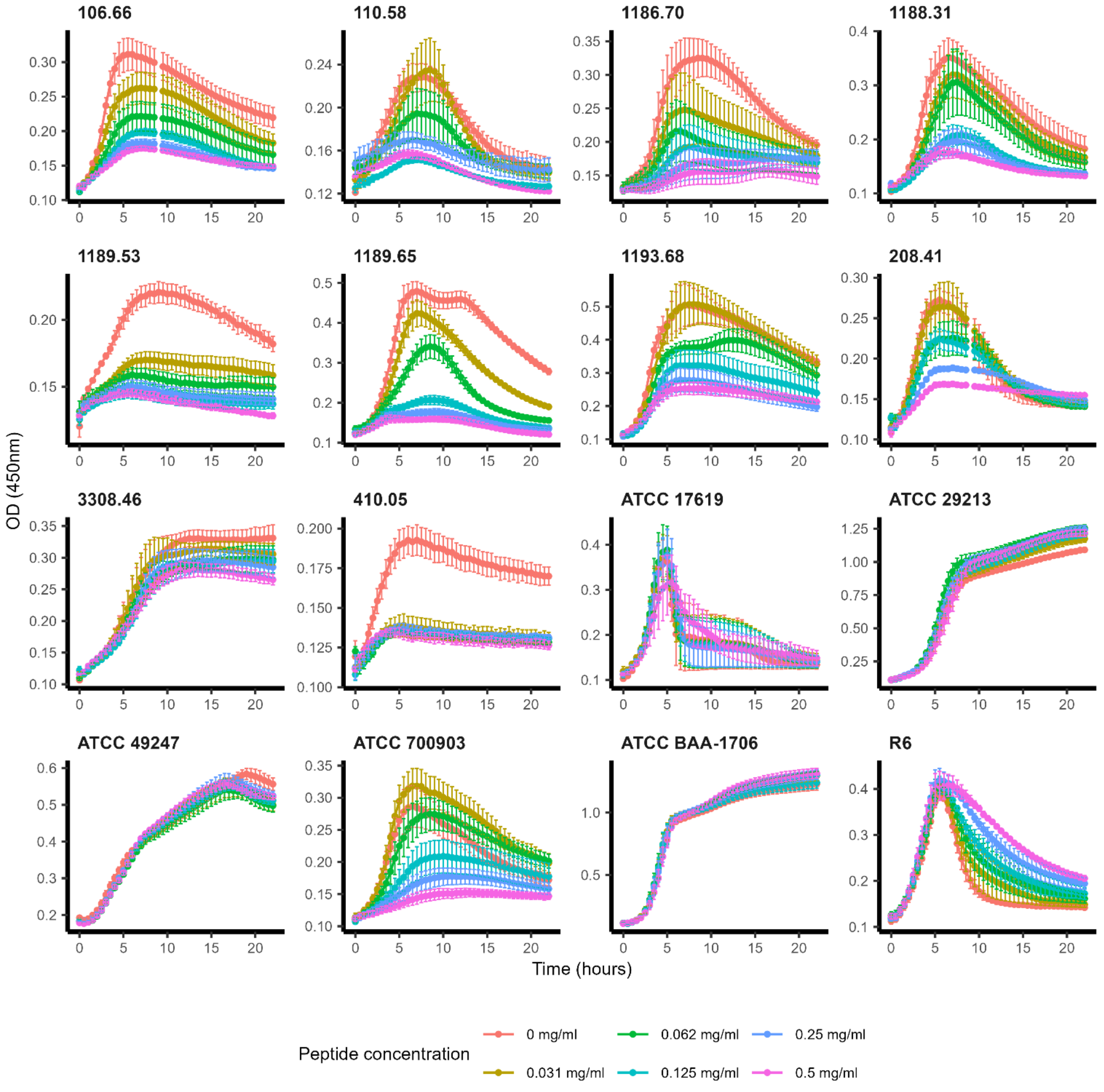

Supplementary Fig. 1: Growth curves of bacterial strains used to calculate MIC_{50} for Table 1. *S. pneumoniae* strains: 106.66, 110.58, 1186.70, 1188.31, 1189.53, 1189.65, 1193.68, 208.41, ATCC 17619, ATCC 700903, R6; *S. mitis* strain: 3308.46; *S. pseudopneumoniae* strain: 410.05; *S. aureus* strain: ATCC 29213; *H. influenzae* strain: ATCC 49247; *K. pneumoniae* strain: ATCC BAA-1706.

Growth was measured in peptide-free chemically defined medium (CDM) in presence or absence of peptide V11A by measuring optical density OD (450 nm). Results represent 3 independent experiments, error bars indicate SEM.

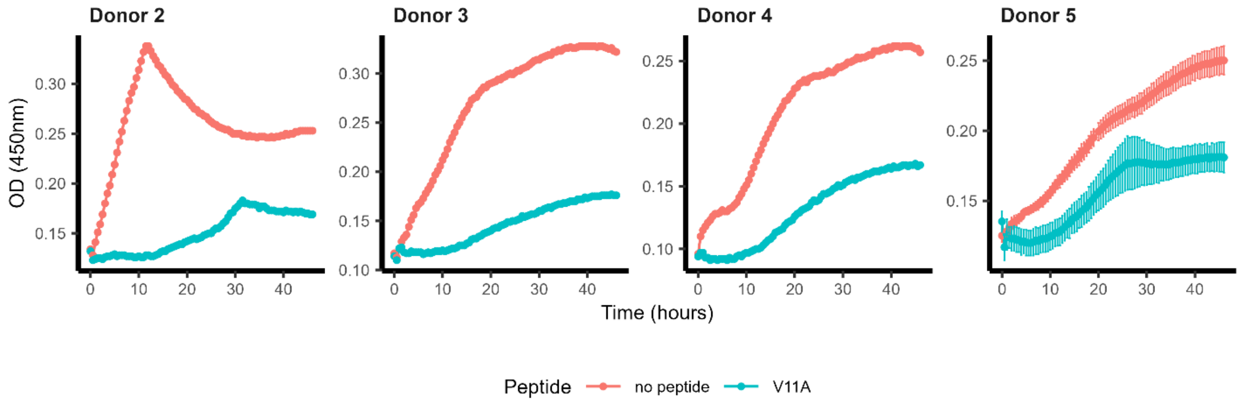

Supplementary Fig. 2: Effect of peptide V11A on pneumococcal growth in human cerebrospinal fluid. Growth curves of trimethoprim-sulfamethoxazole resistant clinical pneumococcal isolate 1154.75 (serotype 23F) in presence and absence of peptide V11A (0.5 mg/ml) in human cerebrospinal fluid (hCSF) of different donors. Growth was measured in undiluted hCSF by measuring optical density OD (450 nm). Results represent 1 technical replicate of the same experiment (1 biological replicate) for donors 2-4 and 3-5 technical replicates from a total of 2 independent experiments (2 biological replicates) from donor 5 due to limited available hCSF, error bars indicate SEM.

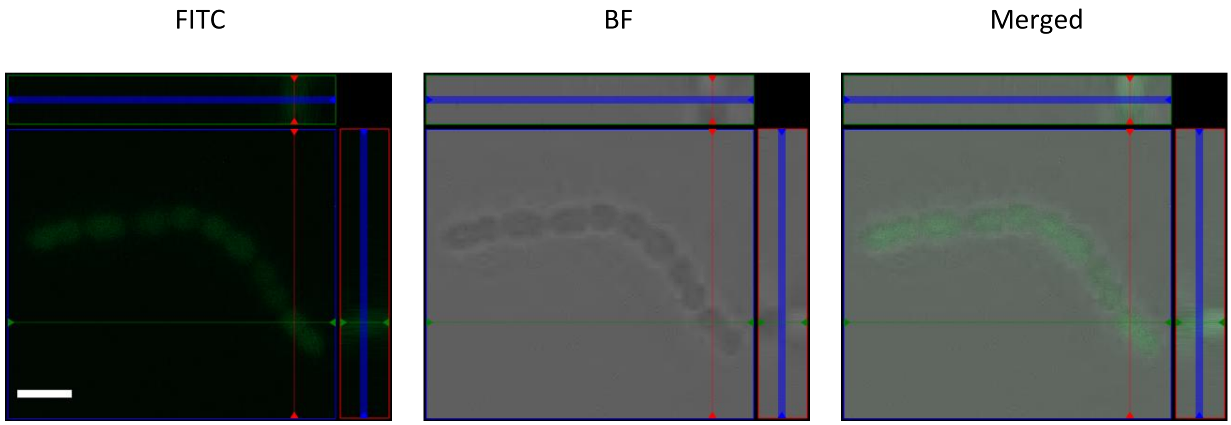

Supplementary Fig. 3: Peptide V11A inside pneumococcal cells of strain D39. Representative images of *S. pneumoniae* strain D39 after incubation with FITC-labelled peptide V11A. Scale bar indicates 2 µm for all pictures. Orthogonal view from z stack to analyse location of FITC-labelled peptide V11A. Colocalization of peptide in FITC channel with cytosol of bacterium in Brightfield (BF) channel.

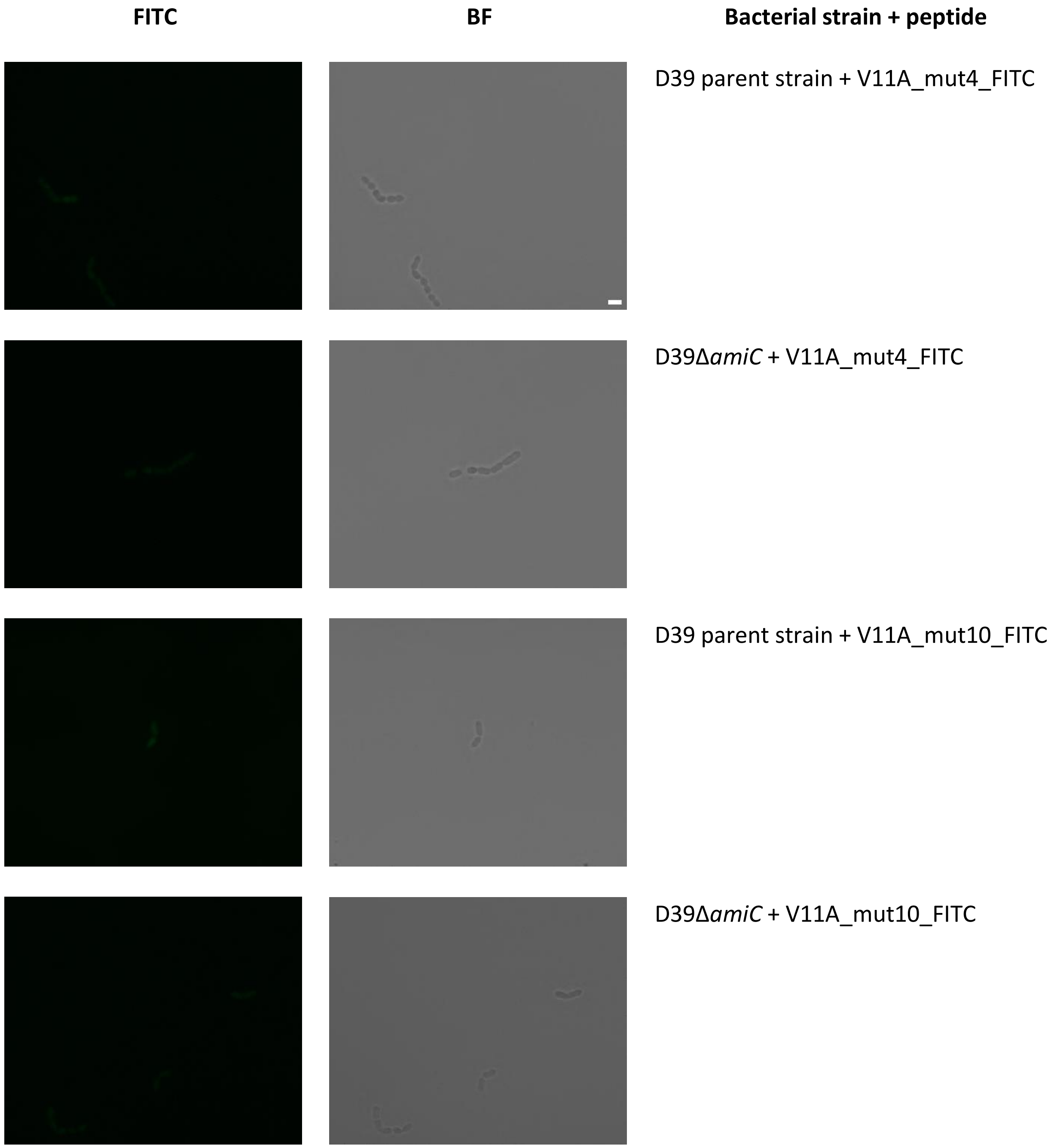

Supplementary Fig. 4: Epifluorescence microscopy of V11A control peptides on pneumococcal cells. Microscopy images of *S. pneumoniae* D39 parent strain and Δ*amiC* mutant after incubation with FITC-labelled control peptides for V11A (V11A with amino acid difference at position 4 or position 10). Representative images were taken at mid-bacterium localization in z position showing FITC and Brightfield (BF) channel. Scale bar indicates 2 µm for all pictures.

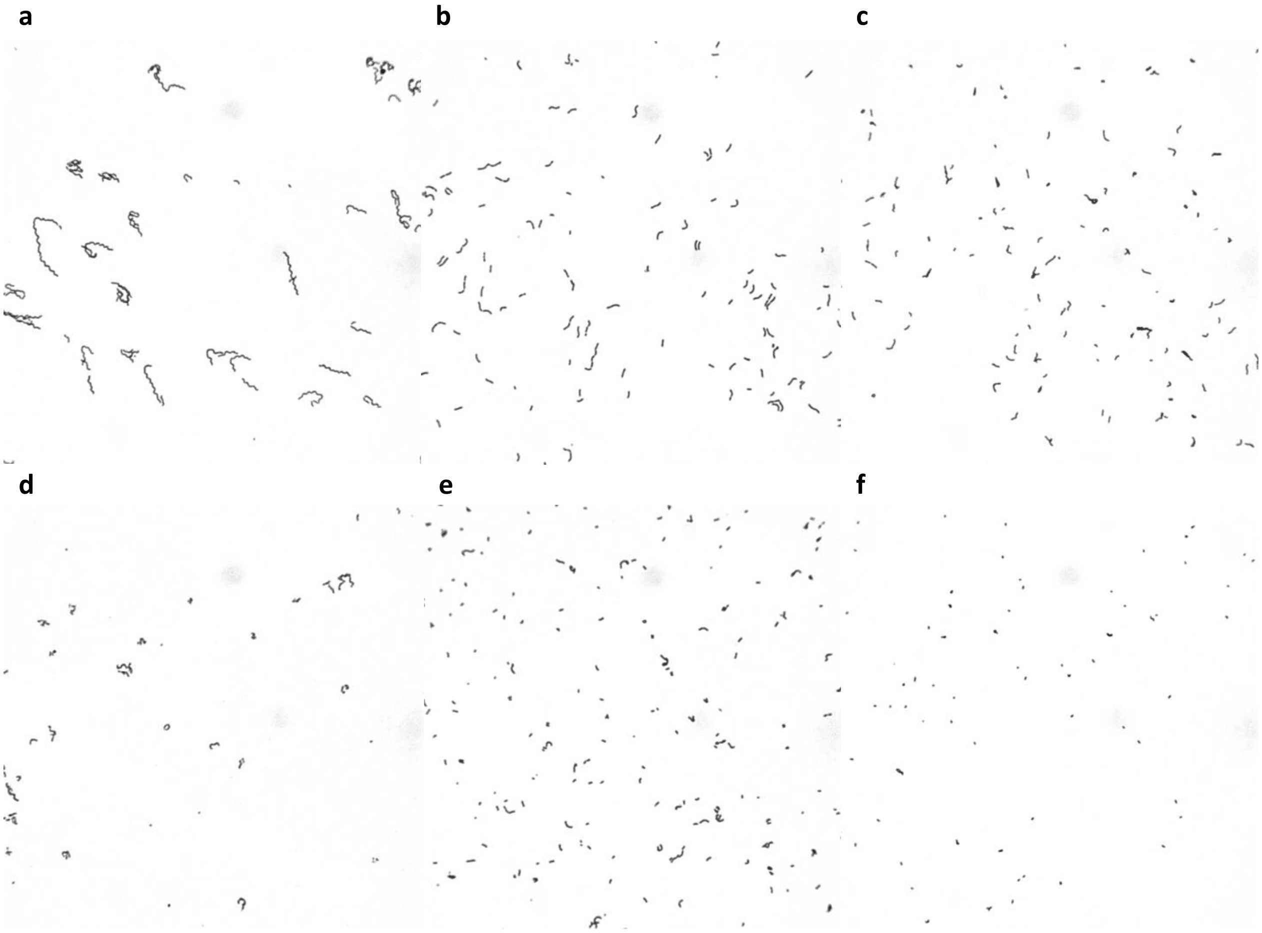

Supplementary Fig. 5: Effect of peptide V11A on pneumococcal chain length. Representative phase contrast pictures show chain length for *S. pneumoniae* strains **(a)** D39, **(b)** 106.66, **(c)** 208.41 without peptide and chain length for **(d)** D39, **(e)** 106.66 and **(f)** 208.41 after incubation with 0.5 mg/ml peptide V11A for 2.5 h. Pictures were taken with 40x objective.

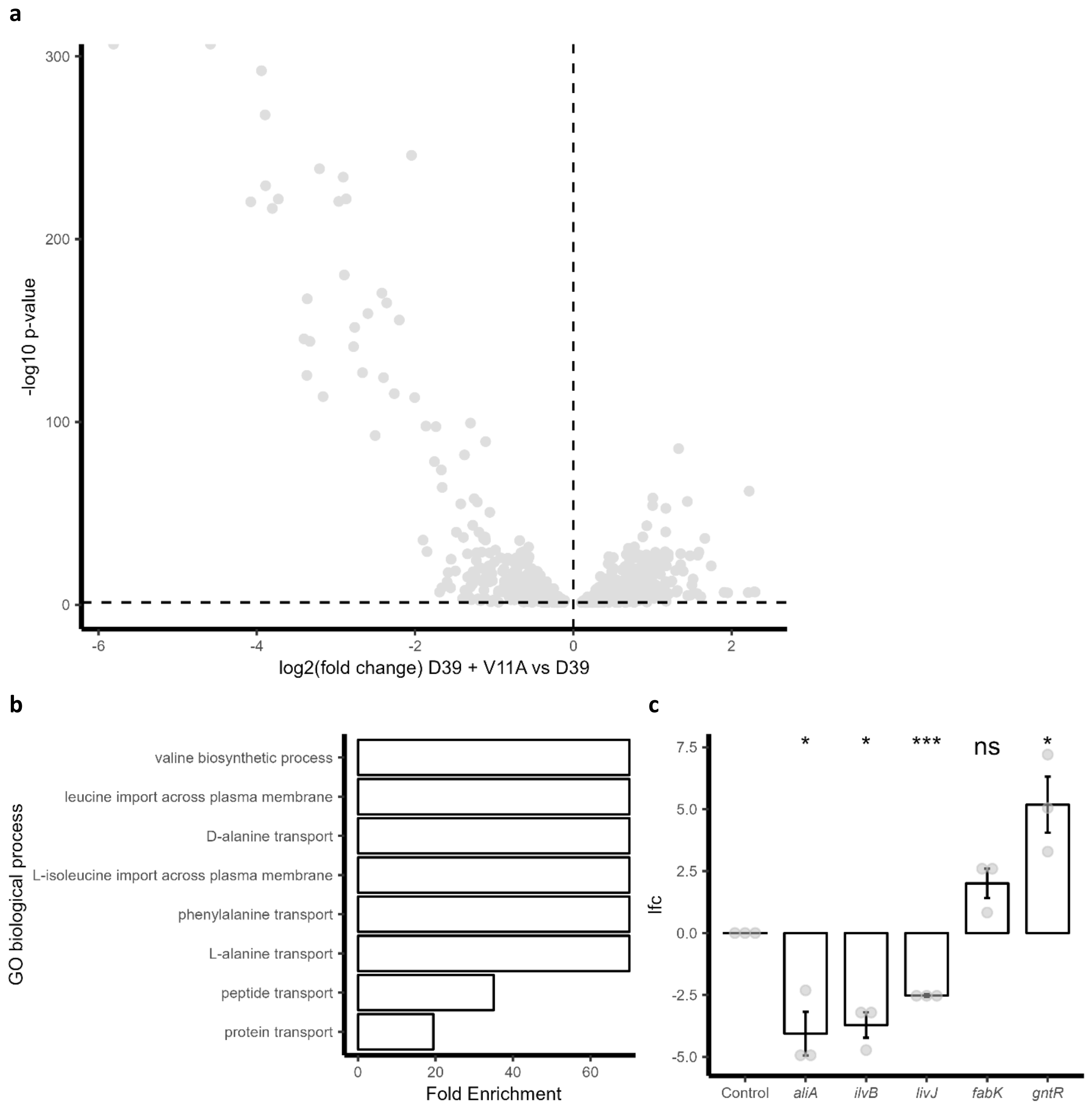

Supplementary Fig. 6: Effect of peptide V11A on pneumococcal transcriptome. (a) Volcano plot showing pneumococcal gene expression changes caused by peptide V11A treatment from RNA-Seq data with 6 biological replicates. **(b)** Gene Ontology biological process enrichment analysis showed enrichment of several protein metabolism and transport processes in genes significantly downregulated after V11A peptide treatment of *S. pneumoniae* D39 with log2FC ≤ -1.25. No GO term enrichment was found in genes significantly upregulated after peptide treatment with log2FC ≥ 1.25. **(c)** Differential gene expression confirmation with real-time RT-PCR. The data represents 3 biological replicates. "ns" indicates

not significant, * indicates p value ≤ 0.05, ***: p value ≤ 0.001 by pairwise t-test. P values are 0.04390 for *aliA*, 0.01849 for *ilvB*, 0.00053 for *livJ*, 0.07709 for *fabK*, 0.04463 for *gntR* (here abbreviation for SPD_1524).

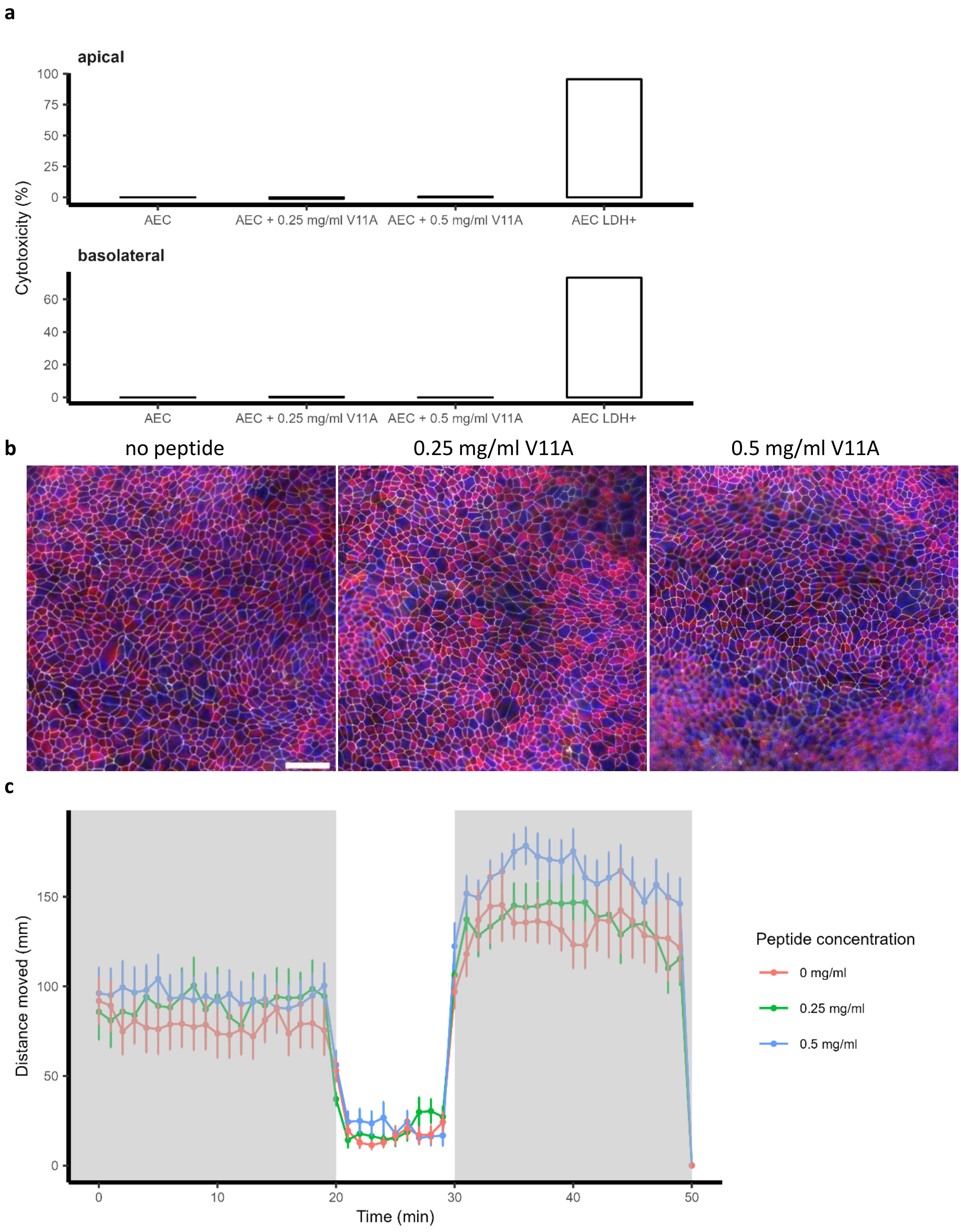

a
apical
100
75
50
25
0
Cytotoxicity (%)
AEC
AEC + 0.25 mg/ml V11A
AEC + 0.5 mg/ml V11A
AEC LDH+
basolateral
60
40
20
0
AEC
AEC + 0.25 mg/ml V11A
AEC + 0.5 mg/ml V11A
AEC LDH+
b
no peptide
0.25 mg/ml V11A
0.5 mg/ml V11A
c
Distance moved (mm)
150
100
50
0
0
10
20
30
40
50
Time (min)
Peptide concentration
0 mg/ml
0.25 mg/ml
0.5 mg/ml

Supplementary Fig. 7: Non-toxicity of peptide V11A on human airway epithelial cell cultures or zebrafish larvae. (a) Lactate dehydrogenase (LDH) release was low in hAEC (human airway epithelial cells) cultures (apical or basolateral side) untreated or treated with peptide V11A for 30 h, indicating no cytotoxicity. LDH+ control was used to assess 100 % cytotoxicity. **(b)** Immunofluorescence of hAEC cultures with or without peptide. AEC cultures without peptide have the same morphology as AEC cultures treated for 30 h with 0.25 mg/ml V11A or 0.5 mg/ml V11A. The images were taken with 40x objective, blue: DAPI for nucleus, red: β-Tubulin IV for ciliated cells, grey: ZO-1 for tight junctions. Scale bar indicates 50 µm for all images. **(c)** Light-dark locomotion test shows similar swimming response to a change in light conditions for zebrafish larvae in presence or absence of peptide V11A. Shading indicates darkness. Distance moved was measured for single larvae of each group in wells of a 96-well plate in a Danio vision observation chamber. Results represent 26-30 larvae per group, error bars indicate SEM.

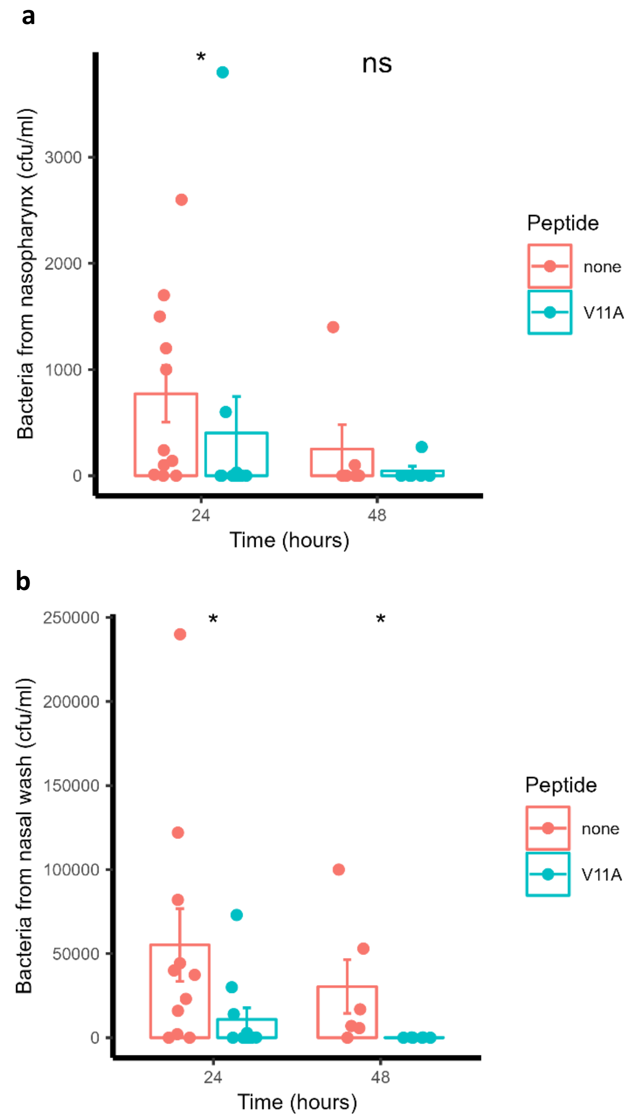

Supplementary Fig. 8: Effect of peptide V11A on pneumococcal colonization in vivo. Peptide V11A (0.5 mg/ml) effect on colonization of nasopharynx **(a)** and bacterial load in nasal wash **(b)** of rat pups inoculated with pneumococal clinical isolate 1154.75 (serotype 23F). Bacteria recovered from nasopharynx and in nasal wash were quantified at 24 h and 48 h post inoculation by dilution plating and counting colony forming units (cfu). * indicates p value = 0.01367 in (a)**,** * indicates p value = 0.01989 in (b) for 24 h and 0.01704 for 48 h by pairwise one-sided Wilcoxon rank sum test. Data points of 11 individual pups per condition for 24 h timepoint (from 2 different days with 6 and 5 pups respectively) and 6 individual pups per condition for 48 h timepoint are represented by dots, error bars indicate SEM.

SUPPLEMENTARY TABLES

a

Protein	Gene	Description
**off**		
A0A0H2ZML6	*pnpS*	Histidine kinase
A0A0H2ZNU3	SPD_0968	Acetyltransferase, GNAT family protein
A0A0H2ZNX1	SPD_1307	Uncharacterized protein
A0A0H2ZPG1	SPD_0569	Sodium/hydrogen exchanger family protein
A0A0H2ZPJ6	SPD_1565	Transcriptional regulator, putative
A0A0H2ZPQ8	SPD_0956	ABC transporter, ATP-binding protein
A0A0H2ZPR9	SPD_1518	Transcriptional activator, Rgg/GadR/MutR family protein
Q04JF7	*truA*	tRNA pseudouridine synthase A
Q04KB1	*xseB*	Exodeoxyribonuclease 7 small subunit
Q04KF1	*xerS*	Tyrosine recombinase XerS
**on**		
A0A0H2ZM69	*rnmV*	Ribonuclease M5
A0A0H2ZNA5	SPD_1264	ABC transporter, ATP-binding protein
A0A0H2ZPM4	SPD_0614	ABC transporter, ATP-binding protein

Q04I25	*arcB*	Ornithine carbamoyltransferase

b

Protein	Gene	Description
**off**		
A0A0H2ZM30	*livH*	Branched-chain amino acid ABC transporter, permease protein
A0A0H2ZM74	*rpe*	Ribulose-phosphate 3-epimerase
A0A0H2ZMP2	*ribB*	Riboflavin biosynthesis protein RibBA
A0A0H2ZNL6	SPD_0052	Phosphoribosylformylglycinamidine synthase, putative
A0A0H2ZNX8	SPD_0285	Glycosyl hydrolase, family protein 31
A0A0H2ZPQ1	SPD_1079	Type II restriction endonuclease, putative
A0A0H2ZQZ7	*secE*	Preprotein translocase, SecE subunit
**on**		
Q04KC1	*lacA*	Galactose-6-phosphate isomerase subunit LacA

Supplementary Table 1: Effect of peptide V11A on the pneumococcal proteome. Proteomic changes detected in *S. pneumoniae* strain 106.66 **(a)** and 208.41 **(b)** after 15 min of exposure to 0.5 mg/ml peptide V11A: Proteins that were only detected in the absence of peptide ("off") or only detected in presence of peptide ("on"). Proteins were strictly seen in one group, but not the other.

**Forward Primer Sequence**	**Reverse Primer Sequence**	**Reporter 1 Dye**	**Reporter 1 Sequence**	**Reporter 1 Quencher**	**Assay Name**
TCCTATAAATACACATCTAAGACCAGCGA	CAACTGAGAGGCATAGGCTGTAC	FAM	CCTGACGGAAATCCTT	NFQ	ALIA
GGTAGCCACAGCTGTATCTATTCCT	GCCGCAGCACCTTCAC	FAM	CATCCGCAATTCCTCC	NFQ	FABK
CGAGTCATACAAGGGTGAAATCGTT	ACGATAGCATCAAAGTCTTTCCCTTTC	FAM	CCTGCTACGAAAGTTT	NFQ	LIVJ
TTGGAATGGGAGGCATGCA	TCATAAAGTCCGCTTCCGTCATG	FAM	TCGCAGCAAATATTG	NFQ	ILVB
GACGATACATAGCCGACCTGAGA	GTAGGAGTCTGGGCCGTGTCT	FAM	CCAGTGTGGCCGATC	NFQ	16S
CAACTGGACGATTTGTGACTAAGGA	GTCATGGAGGAAACGAAGTGTTC	FAM	CAGTCACGCAAACAAT	NFQ	GNTR

Supplementary Table 2: Details for Custom TaqMan Gene Expression Assays.
