# Supplementary material for: Klebsiella pneumoniae peptide hijacks a Streptococcus pneumoniae permease to subvert pneumococcal growth and colonization
Source: Commun Biol. 2024 Apr 8;7:425. doi: 10.1038/s42003-024-06113-9 (PMC11001997; doi:10.1038/s42003-024-06113-9)
Supplement: Supplementary file 3 — Description of Additional Supplementary Materials [file 42003_2024_6113_MOESM3_ESM.docx]

**Description of Additional Supplementary Files**

**File name: Supplementary Data 1**

**Description:** The source data behind the graphs in the paper

**File name: Supplementary Data 2**

**Description:** RNA data

**File name: Supplementary Data 3**

**Description:** Proteomic data
